# The historical nature of biological complexity and the ineffectiveness of the mathematical approach to it

**DOI:** 10.1007/s12064-022-00369-7

**Published:** 2022-05-18

**Authors:** Saverio Forestiero

**Affiliations:** 1 Department of Biology, University of Rome “Tor Vergata”, Rome, Italy; 2 Res Viva, Interuniversity Research Center for the Epistemology and History of Life Sciences, Rome, Italy

**Keywords:** Biological complexity, Organization, Individuality, Variation, Biodiversity, Relationality

## Abstract

Contemporary scientific knowledge is built on both methodological and epistemological reductionism. The discovery of the limitations of the reductionist paradigm in the mathematical treatment of certain physical phenomena originated the notion of complexity, both as a pattern and process. After clarifying some very general terms and ideas on biological evolution and biological complexity, the article will tackle to seek to summarize the debate on biological complexity and discuss the difference between complexities of living and inert matter. Some examples of the major successes of mathematics applied to biological problems will follow; the notion of an intrinsic limitation in the application of mathematics to biological complexity as a global, relational, and historical phenomenon at the individual and species level will also be advanced.

## Evolution of living dynamic systems and complexity

Unlike the evolution of the inert matter of the whole universe, consisting of processes of transformation of physical objects which, while presenting different manifestations at different space–time scales of observation and measurement, always remain transformation processes, biological evolution resembles that set of phenomena which have their origin in the variation in biological systems of every order and degree and which in turn produce biological systems still capable of variation. In essence, while the evolution of physical systems occurs by transformation, biological evolution occurs by variation. (Forestiero 2004, 2007). Inherited variation is so to speak the fuel of evolution.

As far as we know, this fundamental distinction between the evolution of these two classes of objects has never been contradicted by scientific research.

We believe that this distinction can be an interesting premise from which to move in order to trace, identify, and summarily describe the minimum set of constitutive features of biological complexity. Living matter is a particular state of matter and follows the laws of physics (Wolf et al. 2018). However, as we will see, it possesses exclusive defining characteristics that produce a hierarchical complexity without equal outside the domain of biology.

Before proceeding with our discussion, let us keep in mind that, within living systems, dysfunctional processes may also occur, procedural errors that can decrease the efficiency of the system and also lead to the loss of its unity, of the relationships between its components, and ultimately to its death: an exclusive feature of living individuals. From a thermodynamic point of view, these procedural errors correspond to an increase in the disorder of the individual system at the expense of order, an increase of entropy at the expense of negentropy. Functional living systems on the other hand use energy in order to maintain their inner order and tend to evolve towards growing levels of order and organization (Schrödinger 1944).

The contradiction between function and dysfunction is one of the many oppositions that characterize living matter, and it is decisive for the very existence of individuals.

Along with that between order and disorder (the living “*Entre le cristal et la fumée*” by Atlan 1979), there is the opposition between stability and instability, variation and its opposite, differentiation and integration, openness (in a thermodynamic sense, with respect to the structure) and closure (with respect to the internal organization), there are the multiple and often antagonistic relationships with time (internal metabolic oscillators, the nictemeral or seasonal rhythms, periodicity or aperiodicity of signals external phase-shifted from the internal rhythms), and many more (Forestiero 1999, 2000).

There are also other distinctions in the relationship between the parts and the whole. In an individual organism, the robustness (resistance to change) of the whole can compensate for the poor efficiency of some component, and, more generally, the efficiency of the mechanisms of the components can be replaced by the characteristic of macroscopic robustness of the whole, as for example during the development processes. In general terms, a biological system is robust if it continues to function in the face of internal noise (mutations) or external (environmental) perturbations (Carlson and Doyle 2002; Kitano 2004; Wagner 2005). As Kitano writes: *It is important to realize that robustness is concerned with maintaining functions of a system rather than system states, which distinguishes robustness from stability* (strictly speaking) *or homeostasis.* [The latter] *… is clearly a property that maintains the state of the system rather than its functions. Homeostasis, stability, and robustness will be identical if the function to be preserved is the one that maintains the state of the system. In addition, the robustness of a subsystem often contributes to homeostasis of the system at the higher level* (Kitano 2007).

This robustness is pervasive at different levels of biological hierarchy, from macromolecules to genetic networks, to whole organisms. But a distinction should be made between the developmental, physiological robustness of an individual organism during its life and the evolutionary robustness of a kind, a class, a typology of individuals, i.e., the production of very similar individuals in spite of mutations, etc.

In terms of structural stability sensu Thom (1975), robustness refers to a systemic property related to a context. Robustness is therefore a relational attitude present at all structural levels of biological (and ecological) hierarchies, as well as in species-specific individual behavior (Bateson and Gluckman 2011). In many cases, robustness correlates with the overall adaptability of a living system.

Let’s us consider embryonic development. This is undoubtedly one of the most complex, harmonious and regulated biological processes, and with its specificities, it represents an exemplary case of how biological complexity manifests itself through robustness (Newman and Forgacs 2005). There are two possible contexts for the appearance of robustness: a neutralist and a selective one (Felix and Wagner 2008); and it is possible that the causal mechanisms may be multiple. Obviously, the neutralist framework is based on the neutral theory of molecular evolution (Kimura 1968, 1983), according to which a large proportion of the genomic differences between the components of a population are due to neutral mutations, i.e., mutations with no effect on the biological fitness of the carriers. The selectionist context is instead explained by Darwinian theory of evolution.

The neutralist context is based on the evidence that the vast majority of biological processes can be carried out according to a huge number of alternative yet equivalent solutions, without affecting individual fitness (Wagner 2005). In practice, many different genotypes will produce only one kind of phenotype.

A second possibility is that robustness is an evolutionary adaptation to perturbations, and this is the hypothesis that interests us here. Let us take for example the development process, in which the embryo is able to remain unchanged when the conditions of the development environment to a certain extent change. This homeostatic capacity, also called regulatory capacity, or canalization (Waddington 1942), the latter concept being difficult to define (Gibson and Wagner 2000), is a capacity that most likely belongs to the entire embryo and not to the individual cells, although these are also recipients of signals potentially capable of changing their fate (Kitano 2004). On the other hand (here is the apparent opposition), the same embryo is capable of changing in relation to changes in the context: it possesses flexibility (Gerhart and Kirschner 1997). Flexibility can refer to the most diverse biological systems: biomolecules, biological organisms, populations, etc.

An example of flexibility at the molecular level is offered by the proteins of the crystalline lens responsible for its refractive power. In general, vertebrate crystallins have been recruited from stress-protective proteins and a number of metabolic enzymes by a gene-sharing mechanism. This cooptation is a fascinating example of evolutionary flexibility in gene use (reviewed by Piatigorsky 1992).

When referring to an individual, flexibility is the ability of the system to generate, by modest mutation, different patterns in different individuals (Kirschner and Gerhart 1998).

Robustness and flexibility coexist in the same developmental system. Furthermore, in the course of cycles of selection for robust, flexible processes suitable for development (and for adult physiology), the evolvability (capacity to generate heritable phenotypic variation) are selected (Kirschner and Gerhart 1998).

Over the last twenty years, robustness, flexibility and evolvability have become highly popular, recurrent terms in biological literature.

Interestingly, robustness and flexibility are system properties that are not specified by any genetic developmental program, they are not the property of the parts, but of the whole, they are global property of huge genotype spaces.

In a way, the coexistence in the same biosystem of two apparently conflicting properties, robustness and flexibility (and from here evolvability) is, if only in part, a situation equivalent to the apparent conflict that arose in physics between the corpuscular and wave nature of light. Faced with contradictions about the nature of light, Bohr considered these contradictions only apparent and resolved them by postulating that the dual aspects are complementary, in a conceptual sense, but also in a material sense (Bohr 1958, 1960). A similar position can be taken with regard to duality, to the existence of a conflict, which is in fact only apparent, between developmental robustness and flexibility-evolvability (Wagner 2008). These two properties are not alternatives, but complementary. One could speak of an opposition if the two properties were both observed and measured, for instance, on the genotype or on the phenotype, but not when they refer one to the genotype, the other to the phenotype. Exploring robustness and evolvability for a specific genotype and phenotype, RNA and its secondary structure, Wagner has shown, for example, that highly robust RNA genotype has low evolvability, while, in contrast, a highly robust phenotype has high evolvability (Wagner 2008). This is not entirely surprising given that any organism is itself an integrated duality of genotype and phenotype, and that moreover, the same phenotype can be produced by many different genotypes (Wagner 2005). Investigating mutational robustness (low production of heritable phenotypic variation) and evolvability (system’s ability to produce heritable variation) has been concluded that robustness enhances evolvability (Wagner 2008). On the other hand, however, if it had not been fully understood for a long time how organisms can be phenotypically robust (to genetic mutations) and yet also can generate the phenotypic variation necessary for evolutionary adaptations, in more recent years, strong indications have emerged that a property of the biosystems known as degeneracy (a partial overlap in the functioning of multi-functional components) plays a central role in the relationships between robustness and evolvability (Whitacre 2010). Moreover, there is evidence that only robustness through degeneracy will lead to evolvability or to hierarchical complexity (see Figure 1 in Whitacre 2010).

At a biomolecular level, computer simulation has clarified a basic and general aspect of the link between a protein’s evolvability and its stability robustness. The models show that the capacity to evolve is enhanced by the mutational robustness conferred by the protein’s extra stability. The latter increases evolvability by allowing a protein to accept a wider range of beneficial mutations while still holding on to its native structure (Bloom et al. 2006).

For a thorough and wide-ranging discussion on evolvability, see Minelli’s updated critical review (Minelli 2017), and Bertolaso et al. (2018) for a recent multi-instrumental exploration of the promoting features of robustness (redudancy, modularity, multiple pathways). In this collection of essays, the articulation between different approaches, methods and languages of biologists, epistemologists, engineers perfectly highlights the value of robustness as a bridge, between the world of natural and artificial systems.

Obviously, mathematical models on system robustness are being produced in ever-increasing numbers. We quote and comment here on just two of them, respectively, Ay and Krakauer (2007) and Ay (2020), whose key points (expressed in verbal language) are clear and comprehensible even for researchers who ignore mathematical formalism.

In the first paper, the authors provide a geometric framework for investigating the robustness of information flows over biological networks. In order to quantify the impact of knockout perturbations on simple networks, information measures are used. In their model, robustness has two components, (a measure of) the causal contribution of a node or nodes, and (a measure of) the change or exclusion dependence, of the network following node removal. In addition to exploring the possible role played by redundancy in increasing robustness, this model investigates also the relationship between robustness measures and related measures of complexity and concludes that robustness always implies a minimal level of complexity.

In the following review (Ay 2020), Ay obviously recognizes the main property of robust systems as the invariance of their function against the removal of some of their structural components. After duly distinguishing the different ways in which biosystems reduce disturbances into two distinct classes: on the one hand, the system’s modeling and control of the environment, and on the other, the system’s intrinsic adaptation to the environment, the author proceeds to an extensive mathematical formalization of the robustness of the second type. Using some of the most recent and cited contributions on this issue, results related to the robustness of function against knockout perturbations are reviewed. The focus on the relationships between robustness, neutrality and adaptation has been maintained.

A final aspect inherent to living beings (as individuals and/or as population-species) to remark on is the existence of negative and positive feedbacks, usually obtained through the iteration of extremely simple procedures, capable however of producing over time patterns of great complexity and generate chaotic phenomena. In biology exponential functions (such as those describing feedback processes) typically dominate otherwise linear functions (Robertson 1991).

The state of the system at time *t* depends on some nonlinear function that describes the state prior to that of observation at time *t*. Likewise, the next generation is somehow a nonlinear function of the previous generation. Feedback is decisive for the very existence of organisms, and it can be so also for the evolution of the species. The growth of a population of bacteria, or any other type of organism, perfectly exemplifies this statement: The growth is initially exponential and approximates logistics when, with the increase in population density, the ecological conditions are changed. It has been shown that feedback, which also acts on the ecological level of biocenosis, is the connecting element between the theory of evolution and chaos theory (May and Oster 1976; May 1979).

The existence of feedback loops is also implicit in theoretical formulations as the following ones: Changes in fitness can cause changes in the frequency distributions of phenotypes, vice versa: changes in the frequency distributions of phenotypes can cause changes in fitness (Robertson and Grant 1996). These results are not surprising, since the circularity of many processes is a constant in the organismal functions.

## Four traits for an identikit of the biological complexity

The greatest weakness of the concept of complexity is the lack of an unambiguous definition (Adami 2002). This weakness can be circumvented, however, if we limit ourselves to an instrumental definition of complexity, keeping in mind that a definition cannot be simple if it has to be that complete.

Living beings are thermodynamically open systems whose internal order is continually threatened by the variability of the ecological parameters on whose values living beings depend. Overcoming environmental constraints, expressed at different spatial and temporal scales, is very probably entrusted by complex nature of the biological systems, at the level of individuals and species.

We will attempt to draw an identikit of biological complexity using four basic features common to all living systems and which identify them as living matter: organization, individuality, variation/diversity, relationality.

### Organization

Biologists recognize that an essential property of all living beings is that they are organized systems (Mayr 1982). To have an organization means presenting a certain series of nonrandom relationships which ensure the inner consistency of the system. The organizational closure corresponds precisely to the autonomy of the system. “*We shall say that autonomous systems are organizationally closed. That is, their organization is characterized by processes such that (1) the processes are related as a network, so that they recursively depend on each other in the generation and realization of the processes themselves, and (2) they constitute the system as a unity recognizable in the space (domain) in which the processes exist.*” (Varela 1979, p. 55).

The organization of living beings (and their developmental and evolutionary processes) is mandated by the control system of the genomic information. For a clear conceptual framework of the principles of genomic regulation, accompanied by examples of the causal chains, see Peter and Davidson (2015).

A system endowed with organization can be considered by an observer as having a purpose. Not, obviously, in the sense that the system has an intentional plan, a design, but rather in the sense, that its behavior can be described as a tendency to reach a stable state of the system. This final state will depend on the characteristics of the system and of the external environment. In order to describe the existence of finality without intentionality, Jacob uses the word “teleonomy” (Jacob 1970), reserving “teleology” for finality associated with intentionality. For a formal definition of Organization, see Atlan (1974).

The decisive aspect of organization has a cybernetic nature. Living beings are objects whose complexity is based on the presence of regulatory mechanisms involving the control of information circulating through the organized living matter. In cybernetic terms, “The complexity of a system is the number of states in which the system can be located or which it can assume,” and the total number of its distinct states is called “variety” (Ashby 1956, p. 57). This information, in turn, in cyber systems (as much as in biological ones?) is bound according to the law of requisite variety (Ashby 1962), which poses limits to the information available in order to generate the internal order of the system in response to the variation in the ecological factors.

In other words, the amount of external perturbations that the system can compensate for is limited by the internal information available. However, for a critical view on Ashby’s law of requisite variety, Jost may be useful (Jost 2021).

Exemplary treatment of the cybernetic nature of the behavior (i.e., if preferable, of the cybernetical approach to the behavior) of living systems has been provided by the zoologist Pietro Omodeo (Omodeo 1979). Especially pertinent to our study is the section in which Omodeo focuses on the mechanisms controlling the flow of information, homeorhesis (in the sense used by Waddington 1957), in space and in regards to development.

The centrality of control and regulation mechanisms in living systems has recently been resumed and discussed in depth by Jost (see below on Relationality), who also highlights the limits of control and regulation devices (Jost 2021). If the survival and reproduction requirements of a living system can be satisfied by resources (matter, energy, information) from the external environment, the system does not need to produce those resources but rather evolve the ability to use them. It follows that any natural living system (as well as quasi-living systems such as viruses, in effect simple informational replicators) lives in an environment that will necessarily be more complex than the system itself.

The “teleonomic” relationships are directly liable for the unitarity of each living system (individual or species) and for its resilience (the tendency of the system to restore initial conditions).

Like robustness, also resilience refers to a set of perturbations (Wagner 2008) and have to do with the generic adaptability of the biological system, but with at least one important difference at the individual level: While resilience has a specifically reactive behavior, robustness is always proactive. Furthermore, resilience refers to the time system takes to recover to its original state, robustness instead can involve devices present at different levels of the systemic organization, guaranteeing functional invariance despite the possible removal (or in any case malfunction) of one or more structural component.

The organization of living beings proves to be of a hierarchical nature by which monocellular organisms, multicellural organisms, populations, and biotic communities present themselves as phenomenic entities characterized by structural configurations and process dynamics that are different at each level and endowed with properties which cannot be immediately traced or predicted.

The taxonomic space of living systems is also characterized in terms of differences in the level of organization, even if a specific definition of organization is lacking and there is no method for measuring it. Among Metazoa, for example, Coelenterata are considered less organized than Anellida and these less than Chordata. In these cases, organization and complexity become interchangeable concepts.

In addition to being hierarchical, organization is also enclosed, thanks to the activation of circularity in mechanisms that would otherwise be linear in terms of cause and effect. The circular logic (a good example of which is given by the causal relationship: gametes-zygotes-gametes) is found at all levels in the hierarchy, from cell to ecosystem.

Living systems are enclosed systems in terms of their inner organization but they are open to the outside world, with which they exchange matter, energy, and information. Each organism is inevitably linked to the environment, and this link is so necessary that it is impossible even conceive of an organism isolated from its environment.

The necessary link between the living system and the environment, the stimuli excercised on the organism, its chances of establishing resilience, all involve the notions of adaptability and adaptation. It has rightly been pointed out that adaptation (see further on) is a relative and cryptoteleological notion (“… *to the existence of the subject to be adapted and the environment to which it is to be adapted*”; Grene 1974). This is true and must be accepted since it is clear that the relational idea and the one which suggests function and functioning are inherent to the notion of organism and organization. They have a descriptive value and cannot be replaced with by different notions.

The relationship between organization and complexity, together with an analysis of many of the special characteristics of living beings, has been widely investigated by Mayr (1982).

### Individuality

Living systems are not repetitive; heterogeneity is the norm. Both structurally and functionally, living systems are distinguishable from non-living natural systems, on the basis of their individuality. As a rule, every living thing has its own uniqueness originally due to the stochastic properties of the source of variation, which is encoded in the genes, translated into the phenotypes, built epigenetically through a sequence of developmental steps, and transmitted to subsequent generations. Within each local population, the sum of individualities translates into the structure of genetic variation by which the population, as a whole, copes with the “pressures” of the environment. The uniqueness of individuals (their qualities) becomes essential for the evolutionary adaptation of the population to a perpetually changing environment.

The vast majority of living systems are made up not only of genetically different entities, but also can be made up of structurally heterogeneous subunits which, therefore, have behaviors that follow different laws from each other. This is the case of the enzyme protein kit of a cell (which catalyzes particular biochemical reactions regulated by specific control mechanisms) or of the different types of constituent cells of the Vertebrate body.

The individuality is a highly complex property. The internal hierarchical structure and the wholeness is maintained by bottom–up and top–down controls. The connections between the parties are ensured mostly by biological relationships.

The individuality of biological systems is a contributing cause and historical product of evolution.

### Variation/diversity

The diversity is the by-product of the individuality and multiplicity of living systems. Globally, biodiversity is made up of all the differences that can be observed in living beings. These differences can be described in terms of quantity, variation or variability of organisms and, more simply, in relation to genes, species, and ecosystems (Heywood and Watson 1995).

Regardless of the scale effects, the diversity of living systems derives from their individuality and contributes to defining their complexity.

In particular, genetic diversity is represented by all the differences between individuals in a population (and between different populations) and can be inherited and recognized at the gene level. This can be traced back, in the end, to the differences in sequence in the basic linkages of nucleic acids. In living beings endowed with sexuality, the genetic innovations resulting from mutations can spread in the population through re-combination, a device which can generate an immense number of differences. The structure and number of chromosomes and the quantity of DNA contained in a cell are examples of genetic diversity. In particular, the quantity of DNA contained in a cell (genome size) makes it possible to compare organisms with a *taxa* level higher than the species level. In bacteria, for example, genome size varies considerably from 6 × 10^5^ bp to more than 10^7^ bp. The genome of mycoplasm is formed by approximately 500 genes, while in other bacteria, the number of genes varies from 500 to 8000. Most eukaryotes have, instead, something like 50,000 exonic genes (the structural genes, i.e., the spliced primary transcripts, are just over 25,000 in our species), and an extremely variable DNA content, from 8.8 × 10^6^ to 6.9 × 10^11^ bp. Sexuality is a mechanism favoring the production of genetic variation, has a huge taxonomic diffusion and can also be viewed as a device for repairing DNA. It is also a mechanism that enables cellular differentiation, the production of genetic variability available to phenotypes for adaptation to environmental changes, a device for increasing cladogenesis and decreasing extinction rates. Basically, sexuality seems to be important because it can act at many levels of organization of living beings, from gene level to population-species level. The taxonomic species diversity, in addition to the spatial, geographical component, has also a time component. The number of species known by science is about 2 million: Those still unknown are estimated to be between 3 and over 30 million (Heywood and Watson 1995). Once completed, the difference producing process in species is irreversible (e.g., Barton 2020).

The ecological differences, and thus, the ecological complexity of ecosystems are the most difficult to define because the outlines of communities and ecosystems are tipically blurred.

Furthermore, it must be borne in mind that ecosystems are not only made up of biological entities, but also include non-biological components: the physical and chemical factors of the habitat. These so-called autoecological parameters are many and they vary in space and time. Temperature, relative humidity, climate, gas concentration, pH of the ambient medium, water availability, salinity, velocity, turbidity, pressure, soil particle size, etc., directly shape the autoecology of the various species and thus influence their synecological relationships. But this is not enough; the description and evaluation of differences at the ecological level must also take into account the spatiotemporal scale factor that in many cases influences the observation-measurement and interpretation of intra- and inter-ecosystem differences. Actually, processes underlying ecological patterns at increasingly larger scales may be different from those (tipically stochastic) at smaller scales, (Allen and Starr 1982; Wiens 1989; Levin 1992; May 1998; Peterson and Parker 1998; Schneider 2001; Wheatley and Johnson 2009; Estes et al. 2018).

The production of overall biodiversity is the result of a very long historical process which began between 3.9 and 3.4 billion years ago, with the beginning of life on Earth.

### Relationality

The maintenance of connections between the living system and its parts, and the outside, its relationality, is a consequence of the thermodynamic opening components of living systems. Unlike in physical systems, functional cohesion among the components of living systems is entrusted above all to signals going from physical systems biological systems, means going from relationships maintained by the action of forces to relationships maintained through signals. The proven possibility for certain non-living systems (for example, the physical system of cells of Bèrnard or the reaction of Belousov and Zhabotinski) of going spontaneously from conditions of chaos to conditions of order has led many researchers to believe that similar processes were responsible for the complexity of living systems and its increment. The analogy between complex systems of a physical or chemical kind and biological ones is, however, only superficial (Ageno 1986). Indeed, the action of external physical forces that tend to organize chaotic units in a molecular chemical system is lacking in biological systems. In these, instead, the element of order, which can organize the heterogeneous components, is represented by the relationships which promote interconnection between the parts (Ageno 1986). These relationships take the shape of a signal. Through signals, the cells in a bacterial culture or the components of a multicellular system transfer information to each other, coordinating the inner processes with what happens externally. At the multicellular level, the system represented by the signal and its receptor not only ensure integration between the various cells (for example during development processes in animals; Bonner 1984), but also make the social integration of individuals possible through the action of hormones or neurotransmitters. Within the groups of organisms, there is a diversification of the signal-receptor couple (Bonner 1984, 1988). This evolutionary diversification produces both a complicating of the system and a compartimentalization, with the placement of cells containing different receptors in some areas of the body. The signals involved in behavior and which are responsible for the production of interspecific premating barriers of reproductive isolation accurately exemplify the function of maintenance and increase in intersystemic diversity. They illustrate, at a very high level of complexity, the nature of the “forces” which produce the dynamics of living systems. In animals endowed with a nervous system and a sufficiently complex brain, the signal-receptor system is still working to enable learning and thus, recursively, to permit the production of more sophisticated mechanisms by which to produce diversity and complexity. And, with reference to another phenomenological context, the ability of living systems to establish relationships is involved also in the epigenetic elaboration of genomic information in processes of immunity recognition, morphogenesis, and ontogeny of behavior. The capacity of living systems to establish relationships with the environment, with the outside world (adaptability), is part of the larger concept of relationality. It must be emphasized that the most important phase of relationality is bio-complexity, which is the product of the interaction between the self and the non-self. For this reason, it is our view that the notion of complexity in living system must not be limited to individual biological system itself but must include the relationships between the system (self) and its environment (non-self). Living systems cannot be investigated as static objects, as non-historical entities but as dynamic systems connected with the continuously changing context of the environment. It took many decades since the birth of scientific ecology before this critical approach to the notion of the environment began to develop (Brandon 1992).

I state that I will not deal here below with the well known notion of environment-*Umwelt* by von Uexküll because it is in a sharp contrast with the Darwinian vision of the environment and its role in evolution. The Estonian zoologist hypothesized that each living being was surrounded by an environment perceived in a subjective way (von Uexküll 1909), the *Umwelt*. The reasoning behind *Umwelt*'s idea starts from the “meaning” attributed by a species to the objects of external reality (von Uexküll 1940). Umwelt idea it does not match the notion of environment as we have defined it so far. The *Umwelt,* therefore, is not the “non-self” but it becomes quite a characteristic of the individual. According to von Uexküll, the species, the individuals of which it is composed, continually create their own *Umwelten*. On the contrary, according to the ecological and evolutionary thought established following the Synthetic Theory of Evolution, all species are formed and transformed mainly due to the action of the surrounding environment on natural populations. Hence the incompatibility between the Darwinian and von Uexküll approaches to the concept of the environment (for a more in-depth discussion, Forestiero 2009).

The history of the conceptualization of the notion of the environment, which began with the population biology mathematical models of the 1960s, shows that biologists and ecologists have resorted to an increasingly refined view of what the environment is, within which context the many facets of scale problems and perceptive-descriptive characterization could be studied (Levins 1968). From environment tout-court, the environment has become homogeneous or heterogeneous, in terms of space; stable or variable, in terms of time; fine-grained or coarse-grained, in regards of exploitation of the resources; predictable or unpredictable, mosaic-shaped or patchy, etc., the variation in ecological parameters, mosaic-shaped or patchy, etc., in regards to the variation in ecological parameters (Forestiero 2009).

The fact that, from a thermodynamic standpoint, living systems are systems open to the outside, which strongly affects their complexity. Examining a living system in order to examine its complexity without considering its context is a highly questionable practice, in terms of methodology as well as from an epistemological standpoint. Isolating the system from a context that explains most of its structure and functioning leads to an irrecoverable loss of information about the system itself. Information about the ecological world is needed by the living system not only to maintain its link with the outside environment, by adapting its inner state, but is essential also for observers to wish to describe and explain its patterns and processes.

In a very recent paper on this journal, published after the first draft of this paragraph, Jurgen Jost addressed many of the topics discussed so far. His core thesis can be summarized here in a few points: (1) the key feature of life is that a biological process can control and regulate other processes, (2) there may be a hierarchy in the control devices, (3) the information used by the system concerns the control of processes and not their content; namely: relevant information is only what is needed for regulation and control, (4) each biological process draws upon the complexity of its environment (Jost 2021).

While I fully agree on all these points, I find the reference to the environment (fourth point) really necessary. Upon now, in fact, it has rarely been highlighted in the literature that the greatest complexity lies in the environment, and that the living system draws on it, extracting in due course matter, energy and information.

Examining the literature on complexity, indeed it is astonishing to discover that, as a rule, the two ubiquitous components of complexity, environment and adaptation, are almost always absent even in the definition of what a living thing is.

## Physical and biological complexities, a comparison

In the scientific literature on complexity, the modern definition and treatment of complexity is traditionally associated with Herbert Simon according to whom “*systems in which the whole is greater than the sum of the parts are complex systems*” (Simon 1962, 1996). There are natural and artificial complex systems. The differences between artificial and biological systems (however, similar in that they share the property of owning a purpose or a function) have been clarified by addressing the problem of the decomposition of a complex system through an in-depth analysis of the relationships among descriptive complexity, interactional complexity, functional organization, and the Simonian idea of near-decomposeability (Wimsatt 1974). For our purposes, it may be useful to draw a brief comparison between the complexity of physical systems—a disorganized complexity (Weaver 1948)—and the organized and auto-reproducible complexity of biological systems where a great number of variables are inter-related in complicated ways.

All natural complex systems, physical and biological, are characterized by the existence of emerging properties and nonlinear interactions between their components.

It is possible to argue that the difference between a complex physical system and a biological system lies in the fact that the physical dynamic system, having fixed the external conditions, always maintains the same properties while the biological system modifies them over time.

Changes in living beings (both individuals and species) occur because their internal organization can change, not only in response to change in ecological factors but also from one generation to the next. In the first case, the modification in the organization concerns the individual who, within certain limits, can modify its morphology, its physiology and its behavior through the regulatory process called ecological adaptation. In the second case, (thanks to reproduction, an exclusive property of biological systems, during which genetic mutations arise resulting in hereditary phenotypic modifications), the change of the organization concerns, first of all, the genetic information of the population-species causing its evolution.

According to this point of view, the “necessarily” complex natural systems are not so much the physical systems as the living ones, the ones that necessarily change both within generations and from one generation to the other.

The literature on complex systems shows that complexity began to be treated mathematically only at the end of the nineteenth century (Poincaré, 1890; Rossi 2010). But it was only later, the same tools and methods used for the physics were applied to biological systems (Kedem and Katchalsky 1958, 1961). In this regard, it should kept in mind that while simple and complex systems coexist in the world of physics, in the living world all observables are always complex: Complexity is I other words necessary and intrinsic property of all biological systems.

I need to stress this last point. Living beings are systems which are thermodynamically in stationary, non-equilibrium states (Prigogine 1968). Unlike physical systems, they are open systems that exchange matter, energy, and information with the environment (Omodeo 1996, 2003, 2008). The set of physical–chemical and biological characteristics that make up the environment of a species does not remain constant over time and unchanged in space. The environment changes continuously in some of its ecological factors. The individuals (or the species) can maintain the functional connection with their living environment only thanks to their biological plasticity (Griffiths and Gray 2002), that is, thanks to their ability to occupy states that are different from each other in the multidimensional space of the phases in which the system can be found. This phase space is defined starting from the initial conditions in which the system was located and on the basis of the previous ecological (individual) or evolutionary (species) path up to the moment when the system is observed.

Since biological systems are always characterized by a great variety, in the cybernetic sense, it is evident that biological complexity must perform a function crucial to the existence of life. One possibility is that complexity is used by living beings to cope with changes in their environment.

A second observation to be made, connected to the previous one, is that all the possible states in which the system can be found are not equally probable. Living system are never isolated; they must constantly exchange of matter, energy, and information with the environment. In other words, in the temporal dynamics (ontogenesis and evolution) of a living being, the phase space is necessarily linked to its material structure, genome, initial conditions, and past history, so that the observable phase space is necessarily always under-saturated with respect to that one expected on the basis of the free play of possibilities. For a living system, a given state (one described through a definite structure, a morphology, a certain physiology, expressing a specific behavior, performing a certain function in a specific biocenosis belonging to a specific ecosystem, etc.) is not equivalent to another; it cannot be exchanged for another. That is, living systems cannot be conceived as ergodic systems. They are objects whose complexity is based on the presence of regulatory mechanisms involving the control of information circulating through the organized living matter. This information, in turn, is bound according to the law of requisite variety (Ashby 1962), which poses limits to the information available in order to generate the internal order of the system in response to the variation in the ecological factors. In other words, the amount of external perturbations that the system can compensate for is limited by the internal information available.

A result of the complex nature of biological systems is the ability to overcome environmental constraints, expressed at different spatial and temporal scales. This is what we call biological adaptation: physiological adaptation when performed by the individual, evolutionary adaptation, when obtained by the species through natural selection.

The reaction of a living system (individual or species) to the unpredictability of environmental changes, that is, adaptation, is in short most likely a consequence of biological complexity. It is, therefore, not unreasonable to view the complexity of living systems as an efficient solution in dealing with the uncertainty of the environment (Wagensberg 2000). As Carlson and Doyle remind us: “*Simple bacteria with several hundred genes, like mycoplasma, require carefully controlled environments, whereas Escherichia coli, with almost 10 times the number of genes, can survive in highly fluctuating environments*” (Carlson and Doyle 2002, p. 2539). Physical systems can be complex, but biological systems must be complex.

## Simplifying the complexity?

Among the many methodological points which we can only hint at, there is the question of simplification. We tend to forget that in biological research, there is usually a large difference between the evidence obtained in vitro and those obtained in vivo, as well as between the results obtained through laboratory versus field studies. We are all aware of how crucial simplification has been for the advancement of the natural sciences. Yet we should be wart that the study of living beings as a whole does not lend itself easily to simplification. In the next paragraph, I will present two examples of simplification which in my opinion are characterized by methodological errors.

We know that is questionable to study living systems in isolation from the environment, as is too often done. In the biology of whole organisms, relations with the environment cannot be ignored, as can be done in classical physics. By ignoring friction, or by reasoning about a pendulum suspended from an inextensible thread, physicists, thanks to this fiction, have translated natural phenomena into symbolic forms, deriving laws of universal significance. In the case of biology, however, these simplifications are not always acceptable. We should be especially careful in the case of species living in changing environments, i.e., diversified in space and time, which are characterized by greater complexity compared to those than living in relatively homogeneous and stable environments.

The second example concerns a different kind of simplification, which is becoming increasingly common: the reduction in biological evolution to the evolution of genes, genotypes, genomes. Although the evolution of genetic information is a sine quanon of evolution, this reduction in which living beings are made to coincide with their DNA is completely arbitrary: an essentialist’s view on the living beings.

The evolution of genomes is only one aspect of the evolutionary dynamics of organisms and, even if its knowledge is enormously more advanced than the knowledge of the production and evolution of phenotypes, this knowledge by no means exhausts the evolutionary phenomenology, which, as a consequence of biological complexity, is hierarchical and multidimensional. The hierarchical nature of the living can be overlooked nor the fact that the thermodynamic aperture is a phenotypic and not a genotypic property. A knowledge of the evolution of genotypes does not explain the reasons for evolution (why are species made as they are made? why do they exhibit certain behaviors? what is the function of certain structures? What determines the extinction of species? etc.), nor does it answer questions about the ecology of phenotypes, of the living beings which structure ecosystems. This is a point to be considered with caution and attention. In ecosystems, there are phenotypes at work, not genotypes. The environment directly affects the dynamics of phenotypes and only secondarily and very indirectly the dynamics of genetic matter. Biological evolution is an enormously more complex phenomenon than the evolution of genes and genomes. Genomes are material memories, memories indispensable to phenotypes, able to cross the generations; but at each generation, the genomes remain and can replicate only if they are embodied in the phenotypes that reproduce, and only if they inhabit the complex organic bodies that act in the ecological theaters of the biosphere (Hutchinson 1965).

And it is not by chance that phenotypes and not genotypes are the ones characterized by the greatest complexity.

## Mathematics and biology: successes and failures

Mathematics comes in biology in two different ways: first and foremost in quantitative and computational form and, secondly, as an attempt to analyze the relationships existing in living systems. Mathematically, biological phenomena have been treated both from a deterministic point of view and from a statistical one. The inductive procedures of experimental biologists make intense use of statistical methodology on a probabilistic basis both in the planning phase of the experiments and in the processing and interpretation of the data. Inference, correlation, association, uni- and multivariate analysis techniques, etc., are all common notions and well-known procedures in biological research. In taxonomic problems, phylogenetic reconstructions, systematics, cell and molecular biology, immunology, epidemiology, neuroscience, physiology, genetics, ecology, ethology, applied biology, etc., mathematics is omnipresent. And let’s not forget biophysics. Obviously, in the last half century, mathematics has become pervasive in biology also through computer science, bioinformatics, simulation, and increasingly in complex modeling.

The reference to some key episodes of the nineteenth and twentieth centuries’ history of some sectors of biology can shed light on the power of mathematics when biological research faces particular, circumscribed, specific problems.

Mathematics entered biology with Mendel. Mendel wanted to produce a mathematical model of the transmission of hereditary characters from parents to children.

During his university studies in Wien (1851–1854), Mendel had attended the experimental physics course held by Christian Doppler and he had a good grasp of the method of scientific research. Doppler taught him also that an experiment can only resolve a problem if it is designed to answer clearly formulated, simple questions and if it is carefully planned, meticulously prepared, and precisely executed (Klein and Klein 2013). Mendel’s exposure to Doppler and other instructors at the Physical Institute made him familiar with the mathematical analysis of natural events (Edelson 1999, p. 35). He studied the statistical principles of meteorology and received mathematical training studying the combinatorial analysis text by Andreas von Ettingshausen, physicist and mathematician of the University of Vienna. Mendel’s familiarity with combinatorics helped him greatly in shaping the results of his hybridization experiments mathematically. A decade later, while conducting experiments on the hybridization of pea plants in the experimental garden of the Monastery of Brünn (Moravia), Mendel sought to find a model that would account for “… *the number of different forms under which the offspring manifest themselves of hybrids…*” (Olby 1966, p. 112). He found the answer by establishing a parallel between hybrid variation and binomial equation, through which he was able to describe the manifestations of hybrid progeny in numerical relations. So far, Mendel’s results did not differ greatly from those achieved decades earlier by Joseph Gottlieb Kölreuter, Carl Friedrich von Gaertner, and William Herbert, three skilled botanical hybridizers. “*What was different was the mathematical analysis that Mendel used on the results, the conclusions he drew from that analysis, and the language he used to describe his conclusions … Small wonder that the nonmathematical plant experts to whom the paper* [Mendel’s 1866 paper on hybridization] *was sent did not grasp its significance.*” (Edelson 1999, p. 37).

Historians of biology agree that if Charles Darwin had known Mendel’s works, published in 1866, and grasped the mathematical significance of his results, Darwin would have rejected the hypothesis of blended inheritance in which he believed. The hypothesis of blended inheritance makes it virtually impossible to explain the persistence of natural variation within populations. Mendel’s work would have offered Darwin a way to effectively respond to the mathematical objections on the loss of variation over the generations advanced by the engineer Fleeming Jenkin from Edinburgh University, which he instead never found a way to do. It took instead 34 years for the discrete Mendelian inheritance model to be accepted and to counter Jenkin’s objections, regarding the maintenance of the recessive variants of a hereditary character in a population over generations, following the so-called rediscovery of Mendel’s in 1900. About which it must be said that a historiographic tradition that seemed entirely well established is surprisingly undergoing modification. Through the detailed study of many sources, including the close correspondence between Armin von Tschermak-Seysenegg and his better-known older brother Erich (who together with Hugo De Vries and Carl Correns was one of Mendel's laws three rediscoverers), historians are reconstructing the fundamental (and hitherto little recognized) role played by Armin in that enterprise. His own experimental research was conducted in Prague with plants and “with the crossbreeding of 5 species of poultry” (Simunek et al 2011). According to Simunek et al. (2011), the young Armin von Tschermak-Seysenegg carried out his experiments when he was already aware of Mendel’s papers. Mendel’s rediscovery therefore has no longer three but four authors: the three well-known botanists and the young Armin von Tschermak-Seysenegg.

Then, it took nearly another decade to get to mathematical papers by G. H. Hardy and W. Weinberg published in 1908. The Cambridge mathematician and the German physician independently each other founded population genetics by introducing the notion of allelic equilibrium in an ideal panmictic population. The Hardy–Weinberg law (in many respects analogous to Newton’s first law) established the existence of a principle of biological inertia according to which, given certain initial conditions in the transition from one generation to another, the frequency of the gene alleles remains unchanged unless at least one of the following conditions is modified: the almost infinite size of the population, the absence of alleles entering and leaving the population, the random crossings, the absence of mutation, the absence of random frequency fluctuations, the absence of selection. The H-W law establishes the constancy of allele frequencies and allows you to calculate the frequency of homozygous and heterozygous genotypes. The mathematical formulation of this simple principle is the theoretical core of population genetics and, therefore, of the ultimate causal explanation of microevolution.

Beginning in the 1920s, the mathematical models connecting genetics and evolution were developed in England by statistician Ronald A. Fisher and biologist John B.S. Haldane, and in the USA by biologist Sewall Wright. Population mathematical genetics had important developments when in the 1940s, these three scientists resorted to the statistical model of evolution, which lead, first on a purely theoretical level and later also at an experimental and observational one**,** to a synthesis of Mendelism with Darwinism and the biometrical approach, culminating in the elaboration of the Synthetic Theory of Evolution (often wrongly called Neodarwinism), the unifying theory on which evolutionary biology has been grounded since then (Provine 1971).

While in population genetics Fisher, Haldane, and Wright extensively used probabilistic techniques, mathematics began to be used in ecology in the form of the deterministic approach of mathematical physics (Kingsland 1995). In the study of biological associations (from the numerical relations between prey and predator up to multispecies interactions, that is, the dynamical behavior of the entire ecological community), Vito Volterra adopted of the tools of classical mechanics, such as the differential equations of infinitesimal analysis (Volterra 1926a, b). The same problems of Volterra were faced by Alfred J. Lotka, who used similar equations and obtained similar solutions, graphically represented by families of curves expressing the demographic fluctuations of the two conflicting populations. Lotka’s reliance on mathematical physics is already evident in the evocative title of his book *Elements of physical biology* (Lotka 1925), later republished as *Elements of mathematical biology* (Lotka 1956). For a collection of seminal papers of the 1920s and 1930s in mathematical ecology (and evolution) by Volterra, Kostitzin, Lotka, Kolmogoroff (Kolmogorov), etc., see Scudo and Ziegler (1978).

In order to test the predictions of the differential equations of Volterra–Lotka, G. F. Gause carried out a series of empirical studies on the dynamics of populations in competition or predation. His results led him to formulate the principle that two species living together cannot occupy the same ecological niche (Gause 1934). This simple, naturalistic, yet previously unheard of principle, (Kingsland 1995), currently known as the “principle of competitive exclusion, or Gause's principle,” later leads to important theoretical advances. Indeed, the experimental contributions inaugurated the modeling approach of quantitative and a historical ecology of G. E. Hutchinson and R. MacArthur, and in particular to the quantitative study of the ecological niche, one of the main concepts of the ecology.

George Evelin Hutchinson defined the ecological niche of a species, its fundamental niche, as a region in a multi-dimensional space shaped by environmental factors that affect the welfare of that species. A niche is “*an n-dimensional hypervolume […], every point in which corresponds to a state of the environment which would permit [a] species […] to exist indefinitely*” (Hutchinson 1957). Mathematization of the ecology boosted with the geographical ecology of R. MacArthur, in which the author assumes the existence of an equilibrium in biocoenoses (MacArthur 1957, 1960). Thanks to its highlighting of the random factors at play and their interactions with deterministic factors, MacArthur’s models, which are not only descriptive but also predictive, offer great advantages. However, they are too simple to be wholly satisfactory. The existence of an equilibrium in biocoenoses (ecological communities) were extended to the island population, with the theory of island equilibrium (MacArthur and Wilson 1963, 1967). This phase of the mathematization of ecology was associated with the 1960s and 1970s project of building a predictive ecological science (Cody and Diamond 1975), focused on the analysis of regularities on a large geographical scale and little influenced by the time factor. A number of studies have come out which, using the equations of Volterra and Lotka on predator–prey mechanisms, have analyzed the limits to similarity and niche width of coexisting species (MacArthur 1972) and the coevolution and character displacement (e.g., Roughgarden 1979; Slatkin 1980). The ambition to construct an ecological theory free of historical connotations is evident when, for example, in MacArthur and Wilson notion that the number of equilibrium species present on an island is controlled by the rate of immigration and the rate of extinction, without any dependence on the biological quality of the species, their previous demographic histories, the way in which the species exploit resources, their ecological value, and so on. In this way, species and their relationships are reduced to mathematically treatable objects. Upon empirical verification, the models seem to work at times, but on other occasions they do not correspond to the actual situations and are therefore inapplicable.

Behind this mathematization of ecology, one can perceive a theoretical drive to find patterns, more than the need to systematize experimental data. The idea is to build an ecology independent of biogeography and the theory of evolution (Deléage 1991), both necessarily marked by the historical events characterizing the evolution of living beings. We may note that, regardless of their limitations, sometimes very severe, ecological and evolutionary mathematical models retain a great “pedagogical” utility for ecologists and evolutionists, who can certainly benefit from being able to think mathematically: making explicit assumptions (usually implicit in verbal models); verifying their effects in the abstract; introducing clarity and transparency into their ideas, concepts and conjectures, and adopting logical rigor, as necessary premises for any productive comparison between hypotheses and empirical facts. On the relationships between biology and mathematics from a general point of view, the recent Jost (2017) can be useful.

## The contribution of geometry to the study of biological complexity: the form-function relationship, epigenetics and adaptation

For over thirty years, aside from the application of equations to biological problems aimed at identifying causal mechanisms, mathematics has intervened only sporadically in functional biology. This holds also for the geometric approach. Through the so-called three-dimensional topology, we are not looking for mechanisms of action, but we go in search for the existence of invariances, with respect to shape and size, associated with the systemic properties detectable during the transformation processes of biological structures. Now, in the centuries-old philosophical tradition, from Aristotle until today, the idea of knowledge-explanation of phenomena (especially natural phenomena) fully corresponds to the idea of a *scire per causas*: The explanation of a phenomenon corresponds to knowing the causes that determine it. By resorting to topology, a philosophical question therefore arises, which, for the moment remains open (Kitcher and Salmon 1989): whether the knowledge of something other than the mechanisms (in our case the invariances) is still true knowledge.

Although, I do believe that to be the case, questions of the theory of explanation are not relevant to the present argument. In any case, philosophical questions related to the theory of explanation are beyond the scope of this paper. I will therefore limit myself to giving an example of the application of topology to functional biology, in the context of the general problem of epigenesis and notably of its effects on the phenomenon of biological adaptation, which, at the micro-evolutionary level, involves the genotype–phenotype relationship.

From a theoretical point of view, probably one of most striking novelty of this century’s research in biology is up to now has been the discovery of certain relationships between the genotype and the phenotype, in relation to how the genotype space is converted in the phenotype space (Lewontin 1992). It is known that the general quantitative relationship between genotype and phenotype for any biological trait is rarely 1:1, but rather 1:many (if not many:many). The same genotype can generate different phenotypes depending first of all on the (environmental) developmental context.

Phenotypic plasticity is now a well-established fact (Gordon 1992; Pigliucci 1996; DeWitt and Scheiner 2004; Pigliucci and Preston 2004). Most likely, plasticity is the norm for most organisms. In any case, the fact remains that phenotypes (of any kind) are more complex than the genotypes that generated them. The problem is to understand how the production of multiple phenotypes is carried out starting from a single genotype; what are the factors, the constraints, that lead to the formation of a given phenotype among all those possible. Ultimately, it is a question of understanding how DNA is translated into individual fitness. Fitness means adaptation and this topic has been the core of evolutionary theory since Darwin until now. So far the mechanism of natural selection (i.e., the differential reproduction of genotypes) has been the only answer to the problem of biological adaptation, keeping in mind that biological adaptation has both an evolutionary dimension and an ecological one (West-Eberhard 1992; Rose and Lauder 1996; Forestiero 2006). Evolutionary adaptation appertains to the population-species level, while the short-term physiological adjustment, the ecological adaptation, is performed at the individual level.

As it is known, although individual adaptation is obviously well studied, it was traditionally seen as a form of physiologically very constrained adaptation and therefore of limited importance for the biological fitness of individuals living in variable environments. Moreover, it was acknowledged that any positive phenotypic modification obtained by the individual throughout its lifetime was not transmitted to the offspring (rejection of the theory of inheritance of acquired characters). The idea was that once the phenotype had been produced, it was editable to a very limited degree. Today, thanks to epigenetics, we know that things are different (Hallgrímsson and Hall 2011). Epigenetics, the study of changes in regulation of gene activity and expression that are not dependent on gene sequences, refers to both heritable changes in gene activity and expression, and also stable, long-term alterations in the transcriptional potential of a cell that are not necessarily heritable.

In the transmission of hereditary data, two different types of information are transmitted: genetic information, such as a DNA sequence, and epigenetic information, such as modifications of the status of the heterochromatin. This condensed form of chromatin is the point. In eukaryotic cells, DNA is bound to histone proteins in an extremely compact structure: the chromatin; nucleosome is the name of the fundamental chromatin unit, repeated n times along the DNA strand. The DNA is arranged around the nucleosome producing a structure with the characteristic appearance of pearls (the nucleosomes on a thread, the first level of chromatin compaction (Sinden 1994)). DNA is twisted hundreds and hundreds of times until it takes on a compact ball shape. Given the length of the DNA, its size and that of the histones, the number and types of twists of the entire structure, the DNA supercoiling produces a geometry of enormous complexity, yet this structure must retain the capacity to unravel to allow the interaction between proteins and DNA when necessary. Heterochromatin remains unchanged during the cell cycle. Condensation produces a domain organization, which prevents the replication machinery from having easy access to DNA. Epigenetic regulation through gene repression can occur with local transcriptional control of DNA linked to individual genes, or with widespread transcription control involving many sequences with extensive chromatin domains. At this point, the problem becomes how to understand the mechanisms governing the interaction between DNA and histone proteins, and the regulation of heterochromatin. It is clear that in epigenetics, the functional unit on which attention should be focused is not the DNA sequence itself, the isolated gene, but the chromosome tract involved in the activity. An in-depth analysis of the topological conditions of DNA that would make cellular events possible (such as the action of topoisomerases which catalyze the winding and relaxation of supercoiled DNA during mitosis for the replication of the molecule) indicates “*the fact that forms possess the capacity to convert dynamically structures and functions one into another*” (Boi 2011, p. 298). In general, many scientists hypothesize that in eukaryotes, the set of epigenetic modifications (e.g., histone modifications, DNA methylation) constitute a set of information that integrates that of the genetic code, forming the epigenetic code of the cell. In his work, Boi tried to provide a topological description of biological systems such as that of chromatin and chromosome in a multilevel and integrative approach. Indeed, from my point of view, Boi’s topological approach would seem to satisfy the demands of a productive approach to the complexity of biological systems. We agree when he writes: “… *simply knowing the parts list of genes and proteins does not tell us much about how life's many biological processes work*”, as well as with his concluding statements: “*one may foresee that a great deal of the future research on the interface between and life sciences will relate to the following* […] *fundamental issues: How did the topology of the double-helix and DNA-proteins complexes evolve and why it is so biologically important for the integrity of cells and organisms*?” "(Boi 2011, pp. 298–299). More generally, with the use of geometry, through the theory of nodes, Boi has managed to examine three-dimensional DNA structures and protein-DNA complexes (Boi 2005, 2007), investigating the complicated events of crossing-over during meiosis. A particularly engaging and elegant work is the one in which he examines the topological and dynamic organization of the nuclear genome and the relationships between the spatial organization of DNA and the chromatin in order “*to know how and why organization influences genes expression and chromosome functions, as well as the emergence of new patterns*” during embryogenesis (Boi 2011).

I would like to emphasize a point: beside the perfectly acceptable choice of research topics located at the crossroads between mathematics and life sciences, I also fully agree with the above method.

It is a non-reductionist method focused on the rules that govern the global and collective behavior of living systems, and a method that allows one to investigate the qualitative properties of systems. It is to be hoped that the encounter between topology and biology can cast light on the fundamental relationship between form and function and therefore help to clarify the most hidden aspects of epigenetic mechanisms at the basis of biological adaptation.

## Some attempts to work out a mathematical theory of life

So far we have pointed out some examples of the extremely extensive range of biological research in which mathematics is present. This research focuses on biological subsystems, the parts of a whole, their approach is reductionist, the treatment quantitative, the purposes descriptive or predictive. On the other hand, as we have already said and will repeat later, despite the widespread use of mathematics in biology, the mathematization of ontogenesis and evolution of species seem to us, to date, an illusion. In the case of geometry, however, we found the focus on the relational, qualitative aspect of the topology very convincing. Precisely because relationality, as we have already seen, is a constitutive attribute of the living; this means that not only atoms and molecules, mass and energy, but also relationships have their own ontology (Bonchev and Rouvray 2005). This is a key point that prompts us to a very brief illustration of the interesting attempts made in the past century to resort to mathematics for the study of the biological complexity of the whole, a mathematics dedicated to the study of the intra and inter-systemic relationships of the living. Let us immediately say that, for various intrinsic and extrinsic research reasons, these attempts appear unsuccessful and have failed to create an established research trend. Not being a mathematician, I have no competence to judge on the matter. The present is therefore only a brief illustration and reflection on this intellectual experience, which I feel can be useful to the present topics.

The above studies were carried out by Nicolas Rashevsky, a Ukrainian mathematician who emigrated to the USA in 1924, and by Robert Rosen, a theoretical biologist who was his best-known student. Although he was not the first mathematician to study biology, Rashevsky was nevertheless the first mathematician to conceive an ambitious research program aimed at the mathematization of biology, to which he contributed with works on cell biology, embryology, neurology and ecology, in addition to developing the first neural network model. Starting from the cybernetic notion of system, and explicitly drawing inspiration from *On growth and form* by D'Arcy Thompson, Rashevsky sought to build “*a systematic mathematical biology, similar in its structure and aims to mathematical physics*” (Fox Keller 2002) through the development of “*Formal tools necessary for the modeling of morphogenesis processes understood as sequences of destabilization and stabilization*” (Turing 1952; Rashevsky 1960; Thom 1968—in Bich 2012). In conceiving his project of a relational biology, Rashevsky adopted general biological principles, such as his “*principle of biological epimorphism*,” which emphasizes qualitative relations as opposed to quantitative aspects, topology instead of metrics (Rashevsky 1954). Following this principle, “*It can be argued that a given biological property* [e.g. metabolism, perception, etc.] *in a higher organism has many more elementary processes than the equivalent biological property of a lower one*. [……]. *The principle is based upon the fact that different organisms can be epimorphically mapped onto each other, after the biological properties were already clearly distinguished and represented. In such epimorphic mappings, the basic relations characterizing the organism as a whole are preserved*. [……]. *Wanting to put his principle into a precise and rational context, he chose topology*” (Hoffmann 2015).

Robert Rosen also studied on relational biology using an approach that is the opposite of the reductionist one, focusing on relationships and functions (Rosen 1970). For Rosen, living beings are material systems which realize certain kind of relational patterns, regardless of the specific materials they are made of. A central aspect of Rosen’s project, which is the core of his study *Life itself* (Rosen 1991), is to understand and describe the way in which the “efficient cause” could be “internalized” in organisms. Rosen based his relational model of living organisms on mathematical mappings. His book’s ambitious message is that a model constructed in this manner can to some degree represent life itself. According to Rosen, an organism (a natural autonomous life form, a self-fabrication system) is characterized by its metabolism (M) and by an incessant activity of repair (R) in certain parts of its system. This self-fabricating incessant activity seeks to forestall a future deleterious internal state. Thus, the living system anticipates deleterious internal changes; self- reference and context dependence are central in the Rosen’s conception of the living being (Rosen 1985a; b). This repair activity explains both evolvability and durability of the organism in the face of phenomena which may oppose it. Rosen’s model was unsuccessful, but critics note that although the specific process of mapping which Rosen built into his model is problematic, (M, R) systems do generate still some of the properties of an organism. Basically, Rosen achieved his purpose only partially; the main concerns were the mathematical aspects of his theory and the simplistic character of the model—unable to account for the complexities of embryogenesis or the process of DNA repair (Cottam et al. 2007).

The models by Rashevsky and Rosen, focused on the relational properties of living systems, on their organization, implies the belief that inert and living matter are divided by a profound gap, and that the methodological model of physics is unsatisfactory when used to describe and explain the living. As we will see, this theme has been taken up with much vigor and originality by more than one contemporary scholar.

## Living matter, biological complexity, evolution

The hierarchical complexity of living organisms can be roughly divided into three different irreducible macro-levels: the biomolecular, organism, and species. The compactness of the evolutionary theory, initially elaborated starting from the species level, and integrated later by the knowledge of genetics and bio-molecular characteristics, has been questioned on the basis of new data and ideas coming especially from the level of the organism. There is currently a strong demand for a revision of the Synthetic Theory of Evolution, a search for an Extended Synthesis (Pigliucci and Müller 2010). As we know, mathematics has contributed, from the beginning, to tackling problems of biomolecular or population nature, but it has had very little weight in regards to individual complexity and the evolution of species. The mathematical approaches to biological complexity seen so far, although of great scientific interest, do not touch upon the problem of higher-order complexity, the complexity of the living as a whole (individual or species). This complexity is marked by its uniqueness. It is a historical complexity, described by the free evolutionary trajectories of the species, badly recorded in the phylogenetic reconstructions of biologists and temporarily frozen in biological adaptations; it is the complexity for which the rule of the part does not apply to the whole. This kind of complexity is intimately connected with living matter.

The differences between inert matter and living matter are well known and have been extensively discussed, also in recent times (e.g., Forestiero 2000; Longo and Montévil 2012). The first and most fundamental difference concerns the repetitiveness of physical objects (all hydrogen atoms are the same) as opposed to the diversity of biological ones (even two monozygotic twins are phenotypically different and the right and left hand of the same person show different dermatoglyphs). This difference between inert matter and living systems carries with it enormous consequences for the mathematical treatment of biological phenomena whenever supramolecular level phenomena occur. On the one hand, living systems are specific (that is, they are individual and historical systems), on the other hand, however, their dynamics, their ontogenetic and evolutionary trajectories are generic (Bailly and Longo 2011). This seems to be the unmovable obstacle that makes invariance mathematics unsuitable for their analysis.

For some time, the questions of the specificity and historicity of living beings and of biology have been addressed, also in a non-reductionist fashion, by scholars with various backgrounds—mathematicians, physicists, biologists, philosophers—who have clarified the main differences between the living world and that of physics, pointing out the reasons for the non-transferability of the mathematical approach developed for physics, to the study of key themes of biology, such as the phenomenon of evolution. Certain innovative aspects of the above research coalesces around the notion of *extended criticality*. By comparing symmetry and criticality in inert and living matter, living matter is considered as a place of “*globally critical phenomena.*” The authors note that in the world of physics, the symmetries are stable and that this invariance is due to the *genericity* of physical objects, together with the *specificity* of their trajectories. By contrast, they highlight that “*in biological situations the relevant theoretical symmetries are not stable, but broken by the temporal flow*” (Longo and Montévil 2011). They therefore hypothesize that biological objects show a specific behavior: “… *their theoretical symmetries change and they become defined / specified along (and by) their history.* […] … *by considering that phase spaces are defined with respect to symmetries, we were lead to the conclusion that there is no stable phase space which would allow to capture or theoretically determine the trajectory of a biological object*” (Bailly and Longo 2011; Longo and Montévil 2012). The most interesting point here is that in living organisms, phenomenic changes in symmetry are not, as in physics instead, circumscribed to specific points in the phase space, but are on the contrary absolutely pervasive. The authors refer to this state of affairs as the existence of an *extended critical transition* (Bailly and Longo 2011; Longo and Montévil 2012, 2013), a *permanent critical transition* (Longo and Montévil 2014). Among the results of these studies, what we are most interested in the recognition of the profound difference between the dynamics of biological systems and physical systems and therefore of the methodological and epistemological irreducibility of biology to physics, as stated above.

## Return to nature: a math of whys?

Some of the question asked by evolutionary biologists cannot be answered by physics or mathematics. These questions are basically the ones that begin with a “why.” Why questions are of the utmost importance in biology because they intercept functional and historical problems, problems associated with Darwinian evolution. Scientific answers to these questions help explain why the biological world of living beings is made the way it is.

For example, why does a particular species of bird have red plumage? Through physiology, chemistry, and physics scientists can explain the mechanisms by which that certain color is produced and how it is perceived. “*The new information introduced by answering “why?” … involves us in the knowing of what causes things to happen independent of the way they happen*” (Mikulecky 2005, p. 105). Physics and mathematical formalism investigate the material causes of the phenomena of the inert world. They can show us how living organisms do what they do but the biological approach goes further, it seeks to know the efficient causes which contribute to producing the world of life as it presents itself. It makes no sense obviously to talk about efficient causes to explain the motion of the planets or a nuclear fission reaction. However, it is essential to look for efficient causes when dealing with functions with the role that a certain biological character plays in the life of an organism. The questions about efficient causes are Darwinian questions which, at least until now, mathematics does not seem to me to have ever formulated. I realize that the functional dimension of living systems is not computable and, therefore, this may be an uninteresting topic for the quantitative approach mathematical, but, as we have seen, attempts have been to deal with issues also using a reductionist scientific approach, such as Newtonian dynamics, by means of topology and relational mathematics (Mikulecky 2005). It is biology, with its scientific questions about whys, that deals with the origin of meaning in living nature all the way up to verbal language and human thought. During evolution, living beings become subjects and objects of mutual significance; with movement and with birth and growing complexity of neuronal networks, new characteristics of animal life emerge; new increasingly complex biological traits, such as cognitive capacity, all the way up to human thought. The manifestations of biological evolution, an open and perennial process, continue.

## Concluding remarks

The question we asked is as follows: Do mathematicians have the tools to deal with the phenomena of biological complexity as a whole, that is, the phenomena of ontogenesis and evolution of living beings as a whole? Despite the highly original and generous attempts of some (e.g., Rashevsky 1954; Rosen 1970, 1991; Thom 1975), in my opinion, the answer is they do not have them, not yet. The history of the natural sciences tells us there have been many cases in which mathematics has shown an extraordinary effectiveness in solving problems related to biological phenomena that are very different from each other. This has happened when a specific problem could be isolated from the general context, when it was unnecessary to take into account the effect of the whole on the parts and the effects of the historical dimension of the living world. In cases in which the phenomenon under investigation manifests itself as a historical product, that is, corresponds to a precise, specific (unnecessary) evolutionary trajectory, whenever we are observing a singularity, whenever we are faced with individuality and variation, mathematics loses its effectiveness.

On page 14 of *Pensieri discreti*, Gian-Carlo Rota writes (Rota 1986, 1993; Kac et al. 1985): “*It is hard to decide whether the lack of real relationship between mathematics and biology is a tragedy, a scandal or a challenge*.” (translation mine).

To this question, everyone will give the answer one prefers. Rota’s doubts prompt me, as an evolutionary biologist, the following questions: Is the limited effectiveness, or in some areas the complete ineffectiveness—of mathematics in biology—to be expected; does it have some intrinsic explanation? Lucid observations on this matter are also found in Longo and Montévil (2011).

Perhaps this question cannot be answered with absolute certainty yet, but in my view, there are many clues that suggest this inadequacy is explained, as we have tried to show, by the different, distinct nature of living beings compared to the objects of the inanimate world. At present, mathematics does not seem capable to tackle biological complexity as I referred to in the present article: biological complexity as a whole.

I would like to stress once more how, to better understand the nature and phenomenology of living beings, it is necessary to shift the focus of investigations from the analysis of individual components to the global relationships among the various subsystems and between system and its environment. The major questions are the context dependence and self-reference of biosystems. It is precisely from the existence of relationships that the new systemic properties, the “emerging properties,” arise, and the knowledge of living beings requires the precise identification, the exact description and a sound explanation-interpretation of these properties in the context of the historical changes of the form-function relationship.

Let us say that even if the book of living nature is also written in mathematical language, I still do not see the signs of the classical Galilean-Newtonian mathematical language, so perfectly suited to the world of inert matter. Perhaps a different mathematical language is needed, one capable of fully grasping and explaining the internal relationships between biological objects and their context: a mathematics centered on the study of relationships. The difference between physics, magnificently innervated by mathematics, and biology is there for all to see: Physics is the axiomatic natural science *par excellence*, where (at least in principle) all laws are deducible from a few first principles, while biology is a historical science, the discipline that studies the living products of the history of our planet. Therefore, it does not study universal entities but historical entities, that is particular, individual, in many respects unnecessary, but the result of a mixture between determinism and probability.

In philosophical terms, my position is a monist one with respect to and full in favor of ontological reductionism, but, at the same time, it is a pluralistic position and contrary to both methodological and epistemological reductionism.

For the moment, some aspects of living matter are best investigated using the conceptual tools of biology; always keeping in mind that ascertained biological regularities are something other than the phenomena explained by the laws of physics and cannot be traced back to them.

Maybe I am hopelessly naïve and certainly I am to ignorant to do anything but hope for it, but it would be such a huge scientific achievement if mathematicians managed to invent a new mathematics tailored for the living beings. After all, this feat was already pulled off once, with post-Newtonian physics, when innovative geometries and mathematics were conceived, making possible the theory of relativity and quantum physics. Be as it may, in recent years, there have been encouraging signs in the academic world of a desire to promote a new, effective synergy between biology and mathematics (Cohen 2004).

Allow me to hope that a new discipline will emerge from this relationship. The future mathematics of the living, in dealing with biological complexity as a whole, will necessarily have to deal with the historical dimension of life, which is cause and effect of its biocomplexity.

What a magnificent challenge for the mathematics to come.
